# A minor high‐grade component in non‐invasive papillary urothelial carcinoma is not associated with a more indolent behaviour

**DOI:** 10.1111/his.70086

**Published:** 2025-12-28

**Authors:** Mina S. Farag, Neda Oghbaei, Jaffar Hussain, André Lametti, Wassim Kassouf, Fadi Brimo

**Affiliations:** ^1^ Department of Pathology McGill University Montreal Quebec Canada; ^2^ Research Institute McGill University Montreal Quebec Canada; ^3^ Department of Urology McGill University Montreal Quebec Canada

**Keywords:** grade, minor, mixed, prognosis, papillary, urothelial

## Abstract

**Aims:**

The latest WHO edition proposed using a cut‐off of ≥5% high‐grade component (%HGc) as a criterion to label non‐invasive papillary urothelial carcinomas as high‐grade (pTaHG). It also suggested that tumours with minor high‐grade component behave more indolently and are better labelled as mixed low‐ and high‐grade papillary carcinomas.

**Methods and Results:**

We investigated the prognostic value of %HGc along with other clinical and morphological parameters. 130 pTaHGs and 96 pTaLGs were included. 9% and 12% of pTaHGs had ≤5% and ≤10% HGc, respectively. Anaplasia was present in six cases (5%), necrosis in 18 cases (14%) and CIS in 5 cases (4%). On average, the highest mitotic count per 1 HPF was 2.4 (range = 0–18), and the mean number of mitoses per 10 HPF was 9 (range: 0–93). The mean tumour diameter was 2.1 cm. Tumour multifocality was observed in 32 cases (25%). Among the histological parameters, only mitotic activity showed a correlation with the %HGc (*P* < 0.001). While recurrence was not significantly different between pTaLGs and pTaHGs (25% vs 35%; *P* = 0.09), stage progression was significantly different (0% vs 8%; *P* = 0.005). The two parameters that were associated with recurrence in pTaHGs were tumour multifocality and BCG therapy, while none was associated with progression. %HGc ≤5% and ≤ 10% did not correlate with lower rates of recurrence nor progression.

**Conclusions:**

Those findings suggest that pTaHGs with minor HGc do not exhibit more indolent behaviour and should not be approached similar to pTaLGs. [Correction added on 13 April 2026, after first online publication: This version changes the preceding sentence from “should be approached” to “should not be approached”.]

## Graphical abstract

Non‐invasive papillary urothelial carcinomas with minor high‐grade component are not associated with a more indolent behavior and should likely be regarded as high‐grade carcinomas.

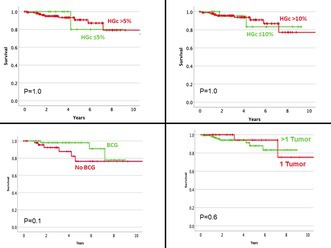

**Abbreviations**
CIS: carcinoma in situ
GUPS: Genitourinary Pathology Society
ISUP: International Society of Urological Pathology
NMIBC: non‐invasive bladder cancers
ROC: receiver operating characteristic
STROBE: Strengthening the Reporting of Observational Studies in Epidemiology
WHO: World Health Organization

## Introduction

Urothelial carcinoma of the bladder is one of the most common malignancies worldwide with most tumours presenting as non‐muscle invasive disease.^1^, ^2^ While most non‐invasive bladder cancers (NMIBC) behave indolently, they are associated with high recurrence rates and a wide range of stage progression, some to muscle‐invasive disease.^3^, ^4^, ^5^ Two of the most important prognostic factors for recurrence and progression of NMIBC are stage and grade, with grade being particularly important in non‐invasive tumours (pTa).^6^ Grading non‐invasive papillary urothelial neoplasms has been a longstanding topic of discussion and debate within the pathology community.^7^, ^8^, ^9^, ^10^ The first bladder cancer grading system for non‐invasive papillary neoplasms was adopted by the World Health Organization (WHO) in 1973, a system in which carcinomas were classified as grade 1, 2, or 3.^7^ This system faced criticism for lacking detailed histologic criteria, which led to the ‘inflation’ of the G2 category. After several attempts, a two‐tier grading system was adopted in 2004, in which carcinomas were classified as low‐grade (pTaLG) and high‐grade (pTaHG), and the term ‘papillary urothelial neoplasm of low‐malignant potential or PUNLMP’ was introduced to prevent the use of the label ‘cancer’ in patients with very low risk of recurrence.^8^ The perceived advantage of the 2004 WHO grading system, which helped its adoption, was the introduction of clear histological definitions for each category. However, while it remains the most widely used grading system among pathologists according to a recent International Society of Urological Pathology (ISUP) survey, it demonstrated several limitations in histological and diagnostic reproducibility, in addition to no added prognostication value.^10^, ^11^, ^12^, ^13^ When grading urothelial papillary lesions, a challenge encountered in daily practice is how to categorize tumours showing mixed low‐ and high‐grade carcinoma components, a phenomenon reported to occur in one third of pTaHGs.^14^, ^15^, ^16^ Until recently, such heterogeneous or mixed tumours (MPUC) were likely and variably labelled as pTaHG by most pathologists, regardless of the percentage of the HGc (%HGc), due to the absence of contrary recommendations. More recently, some studies have shown that a limited HGc does not significantly affect the outcome of such MPUCs, which tended to behave more like pTaLGs rather than pTaHGs.^17^, ^18^ Based on these studies, the latest WHO blue book edition recommended restricting a pTaHG diagnosis to tumours containing ≥5% HGc. Accordingly, the WHO states that tumours with a minor high‐grade component (<5%) are more appropriately labelled as ‘non‐invasive low‐grade papillary urothelial carcinoma with focal high‐grade component’. The Genitourinary Pathology Society (GUPS) also recommends adding a comment to the effect that ‘there is limited data on the prognostic significance of a minor component of high‐grade tumour in an otherwise low‐grade carcinoma, and that studies suggest they generally behave more like low‐grade tumors’.^19^ This recommendation, which may be justifiable at the statistical level based on few pathology‐based studies, has not yet been incorporated into Urology clinical guidelines for managing NMIBC, where pTa tumours are still largely dichotomized into low‐ or high‐grade. Additionally, the term ‘low‐grade with focal high‐grade’ carries an ‘innate ambiguity’ in communication and may be misleading at the clinical level. Without clear guidelines, managing and monitoring such patients remains challenging, particularly regarding the decision to administer (or not) intravesical BCG therapy, whether to conduct upper tract imaging surveillance, and the duration of cystoscopic follow‐up. Interestingly, some more recent studies on this subject have produced conflicting results, which called into question the validity of considering such cases similar to pTaLG tumours.^20^, ^21^ In this context, we decided to systematically investigate the prognostic value of the %HGc component in a cohort of pTaHG tumours.

## Materials and Methods

### Population and Study Design

Patients diagnosed at the McGill University Health Center with de novo pTaHGs between 2015 and 2022 were included. Patients who had a previous diagnosis of pTaHG or pT1HG and those with no follow‐up information were excluded. A control cohort of 96 patients with pTaLGs was included provided patients did not have previous pTaHG/pT1 disease. The TaLG cohort included de‐novo or recurrent cases. The study adhered to the Strengthening the Reporting of Observational Studies in Epidemiology (STROBE) reporting guideline and was conducted according to the principles of the Declaration of Helsinki, and it was approved by the institutional review board.

### Morphologic Characterization and Data Collection

**Figure 1:**
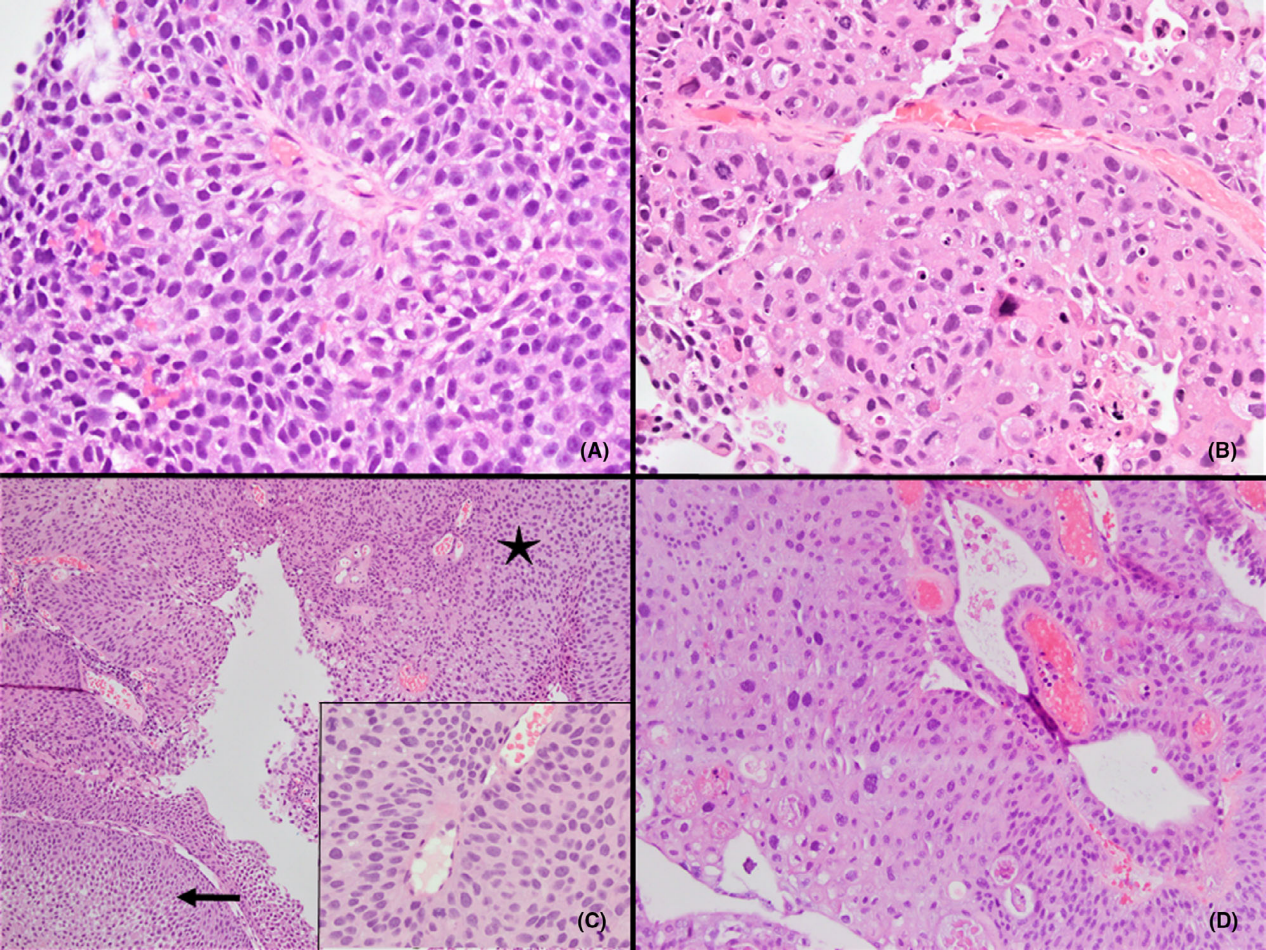
(**A**) Area of high‐grade carcinoma showing loss of polarity, nucleomegaly, severe hyperchromasia, variation in nuclear size, prominent nucleoli and increased mitotic activity. (**B**) area of high‐grade carcinoma showing total loss of polarity, severe nucleomegaly and hyperchromasia, significant pleomorphism and increased mitotic activity with apoptotic bodies. (**C**) Papillary tumour showing areas of low‐grade carcinoma (arrow) with preserved polarity, mild nucleomegaly and moderate hyperchromasia, alternating with foci of high‐grade carcinoma (asterisk), in which the nuclei are not only larger and more hyperchromatic but also more mitotically active with prominent nucleoli. (**D**) Non‐invasive low‐grade papillary urothelial carcinoma with degenerative atypia. The cells on the left are poorly preserved and display smudgy chromatin pattern, falling short of a high‐grade carcinoma diagnosis.

All cases were reviewed by a dedicated Genitourinary pathologist (FB). A high‐grade component was defined as an area with pleomorphic nuclei visible at low to intermediate magnification, exhibiting severe nucleomegaly and severe hyperchromasia. Foci with degenerative‐type atypia were not considered high‐grade (Figure 1). The %HGc was recorded in pTaHGs, along with the following features: (1) anaplasia (defined as high degree of pleomorphism, size variation of more than 5 to 1, bizarre shaped nuclei, generally in a disorganized urothelium); (2) tumour necrosis; (3) carcinoma in situ (CIS), defined as areas of flat epithelium replaced by malignant urothelial cells in tissue fragments that are non‐adjacent and separate from the papillary component; (4) mitotic activity, evaluated as the number of mitoses per 10 HPF and the maximum number of mitoses in a single HPF; (5) presence or absence of muscularis propria; (6) presence or absence of a prominent inverted growth pattern, defined as an inverted component in more than 50% of the tumour, (7) tumour diameter, when available in the cystoscopy report; (8) the number of tissue blocks, serving as an indirect measure of tumour volume. Other clinical parameters included gender, age, body mass index, smoking status and intravesical BCG therapy. Clinical outcomes were derived from both pathology and clinical records (including cystoscopy findings and clinical notes). Recurrence was defined as the reappearance of disease during follow‐up and stage progression was characterized by the emergence of invasive disease (≥pT1) during follow‐up.

### Statistical Analysis

IBM SPSS version 29.0 (IBM Corp, Armonk, NY, USA) was used for statistical analyses. All parameters were tested for normal distribution. Descriptive statistics were used to characterize the entire data set. Associations between variables were examined using Student *t*‐test, *χ* ^2^ test, Mann–Whitney tests, or Wilcoxon signed‐rank tests for continuous variables, and chi‐squared tests or Fisher's exact tests for categorical variables. Pearson correlation coefficients (*r*) and correlation coefficients calculated through multivariate repeated measurement models with a Kronecker product covariance were provided for linear correlations. The performance of the retrieved prediction models was assessed using receiver operating characteristic (ROC) curve analysis. All analyses were performed as two‐sided tests at the 0.05 level of significance. The Kaplan–Meier method was applied to analyse disease progression and recurrence incidence.

## Results

### General Characteristics of the Study Population

This study included 130 patients with de‐novo pTaHG and 96 patients with pTaLG. The median (IQR) follow‐up duration was 3.1 years (range = 1.7–5.1 years) for the pTaHG and 1.5 years (range = 0.1–4.2 years) for the pTaLG cohorts. The demographic and clinical characteristics are detailed in Table 1. Of all pTaHG patients, 56 (43%) underwent BCG treatment.

**Table 1:** Baseline characteristics of TAHG cohort

Variable	TAHG (*n* = 130)
Demographics and clinical characteristics	
Mean age, year (SD)	74.5 (10.1)
Male, *n* (%)	102 (79)
Median FU duration, year (IQR)	3.1 (1.7–5.1)
Treatment with BCG, *n* (%)	56 (43)
Histologic features	
Mean number of blocks (SD)	2.2 (3.3)
Mean number of tumours (SD)	1.5 (1.4)
Mean tumour diameter (SD)	2.1 (2.0)
Pleomorphism/Anaplasia, *n* (%)	6 (5)
Necrosis, *n* (%)	18 (14)
Inverted pattern, *n* (%)	4 (3)
Associated CIS, *n* (%)	5 (4)
Mean highest mitosis per HPF (Range)	2.4 (0–18)
Mean mitosis per 10 HPF (Range)	9 (0–93)
Disease outcomes	
Recurrence	46 (35)
Progression	10 (8)

### Characteristics of High‐Grade Features

**Figure 2:**
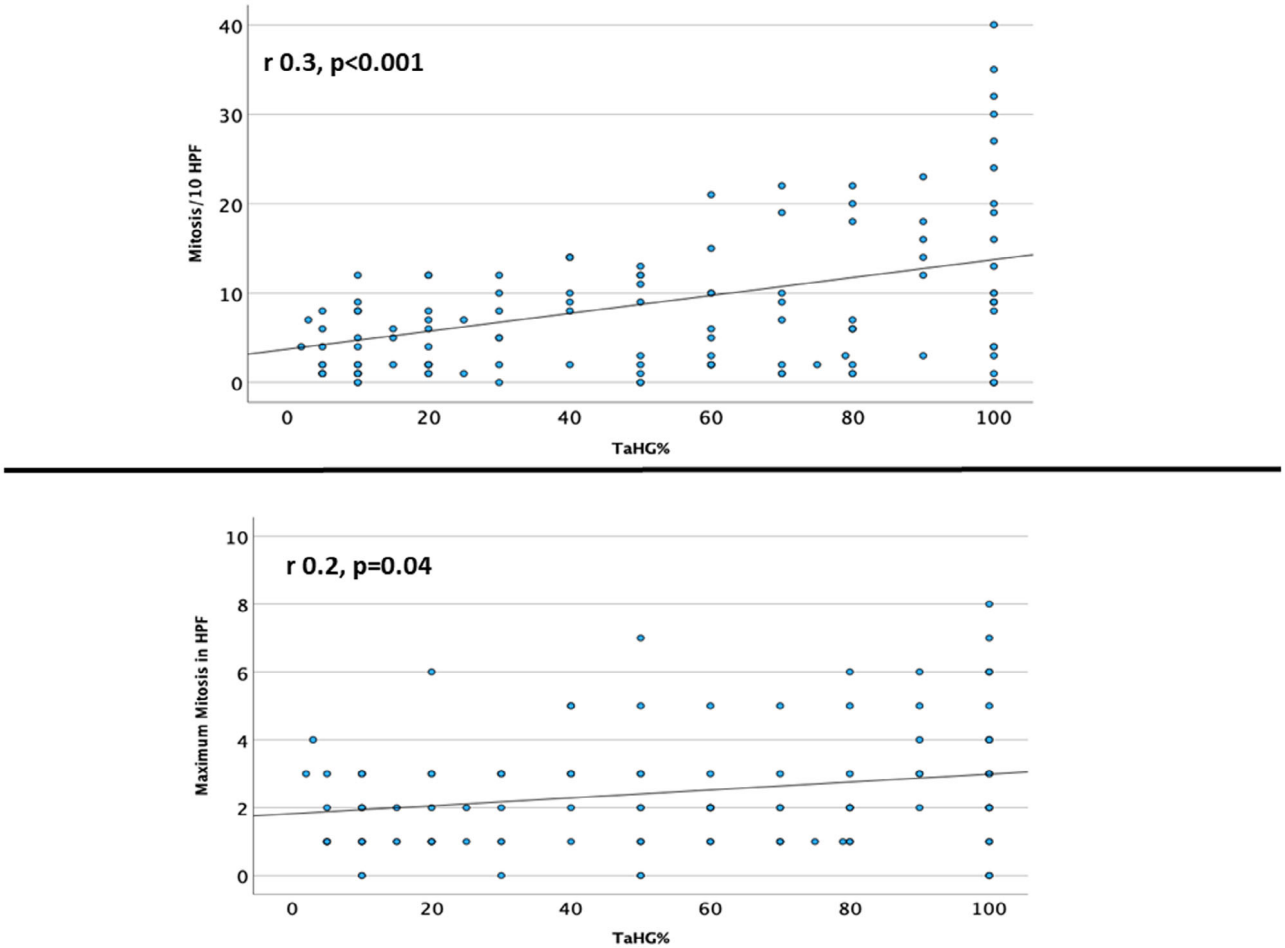
Correlation coefficient of high‐grade component (HGc) and mitotic count, highlighting the linear relationship. (**A**) The correlation of HGc with mitosis count in 10 high‐power fields (HPF), and (**B**) the correlation of HGc with the highest mitotic count in a single HPF.

9% and 12% of cases had %HGc of ≤5% and 5%–10%, respectively. Anaplasia was present in 6 cases (5%), necrosis in 18 (14%) and associated CIS in 5 (4%). On average, the highest mitotic count per 1 HPF was 2.4 (range = 1–18), and the average number of mitoses per 10 HPF was 9 (range: 0–93). The mean (SD) tumour size was 2.1 (2.0) cm. Tumour multifocality was noted in 32 cases (25%). The %HGc was correlated with tumour size, number of blocks, presence of anaplasia, necrosis, inverted pattern, associated CIS and mitotic activity. No significant correlation was noted, except for mitotic activity (*r* = 0.3; *P* < 0.001) (Figure 2).

### Clinical and Histologic Parameters and Disease Outcome

#### Recurrence and progression

The median (IQR) time for recurrence was 3.2 (2.1–5.5) years in pTaHG, and 2.6 (0.7–3.4) years for TaLG. The median time for progression in pTaHG cases was 2.5 (1.2–4.9) years. Recurrence and progression rates for the pTaHG cohort were 35% and 8%, respectively. The only two parameters to be associated with recurrence were tumour multifocality (*P* < 0.001) and BCG therapy (*P* < 0001) (Table 2). None of the histological features nor the %HGc affected recurrence. In comparison, none of the histological nor clinical features correlated with progression. Patients with pTaLG disease had recurrence rate of 25% and no stage progression. While recurrence following a diagnosis of pTaLG was not significantly different from the pTaHG cohort (*P* = 0.09), stage progression was significantly higher in pTaHG patients (*P* = 0.005).

**Table 2:** Correlation between clinical/histologic parameters and outcome

	Progression status		*P*‐value		Recurrence status	*P*‐value
Mean age, year (SD)	Yes	71.5 (6.4)	0.2	Yes	75.0 (10.3)	0.9
	No	74.9 (10.3)		No	74.4 (10.0)	
Gender (male), *n* (%)	Yes	8 (6)	0.9	Yes	37 (29)	0.8
	No	93 (73)		No	64 (50)	
Mean BMI (SD)	Yes	25.3 (3.4)	0.1	Yes	26.1 (4.3)	0.06
	No	27.1 (5.2)		No	27.3 (5.5)	
Current smoking, *n* (%)	Yes	2 (2)	0.3	Yes	6 (5)	0.7
	No	13 (11)		No	9 (7)	
Mean tumour focality (SD)	Yes	1.2 (0.4)	0.2	Yes	2.1 (2.1)	<0.001
	No	1.6 (1.6)		No	1.3 (1.1)	
BCG, *n* (%)	Yes	3 (3)	0.2	Yes	11 (10)	<0.001
	No	53 (50)		No	45 (42)	
Number of blocks, *n* (%)	Yes	2.4 (2.8)	0.5	Yes	1.6 (1.7)	0.07
	No	2.1 (3.2)		No	2.5 (3.7)	
Size ≤3 cm, *n* (%)	Yes	8 (6)	0.9	Yes	41 (32)	0.09
	No	97 (75)		No	64 (50)	
Mean mitosis maximum (SD)	Yes	2.4 (1.6)	0.7	Yes	2.3 (1.9)	0.9
	No	2.4 (2.2)		No	2.5 (2.4)	
Mean mitosis 10 HPF (SD)	Yes	7.2 (5.8)	0.3	Yes	8.0 (9.3)	0.9
	No	9.0 (11.6)		No	9.2 (12.3)	
Necrosis, *n* (%)	Yes	2 (2)	0.6	Yes	9 (7)	0.2
	No	17 (13)		No	10 (8)	
CIS, *n* (%)	Yes	0 (0)	0.4	Yes	2 (2)	0.7
	No	7 (5)		No	5 (4)	
Pleomorphism, *n* (%)	Yes	0 (0)	0.5	Yes	3 (2)	0.5
	No	6 (5)		No	3 (2)	
%HGc, *n* (%)						
≤5	Yes	1 (1)	0.9	Yes	4 (3)	0.9
	No	11 (9)		No	8 (6)	
≤10	Yes	2 (2)	0.9	Yes	11 (9)	0.5
	No	25 (20)		No	16 (12)	

The percentage includes the denominator, which encompasses the entire population.

##### pTAs with minor high‐grade component

Cases with a minor %HGc defined either as ≤5% or ≤ 10% HGc did not demonstrate more indolent behaviour than other pTaHGs. In fact, among cases with progression, one had HGc at 5% and one at 10% with follow‐up periods of 4.23 and 1.79 years, respectively. While there was no significant difference between minor HGc of ≤5% or ≤ 10% and pTaLG in recurrence (*P* = 0.5 and *P* = 0.1, respectively), progression differed significantly between cases with either ≤5% or ≤10% HGc in comparison to pTaLG (*P* = 0.004 and *P* = 0.007, respectively).

**Figure 3:**
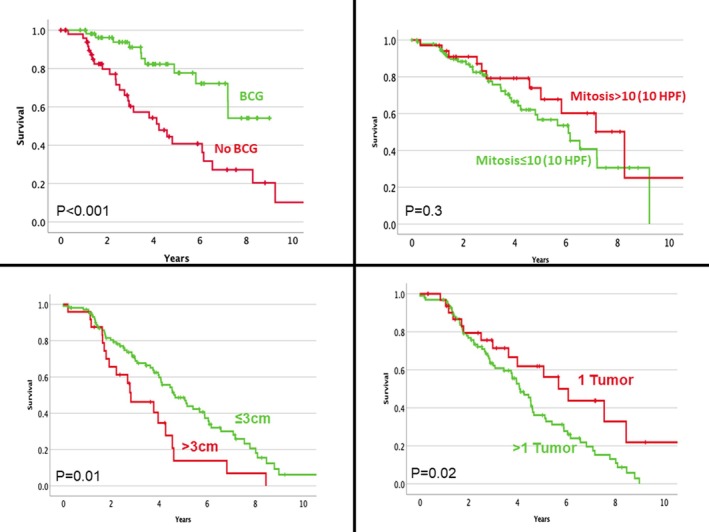
Kaplan–Meier curve illustrating the relationship between various clinical and pathological parameters and recurrence‐free survival.

**Figure 4:**
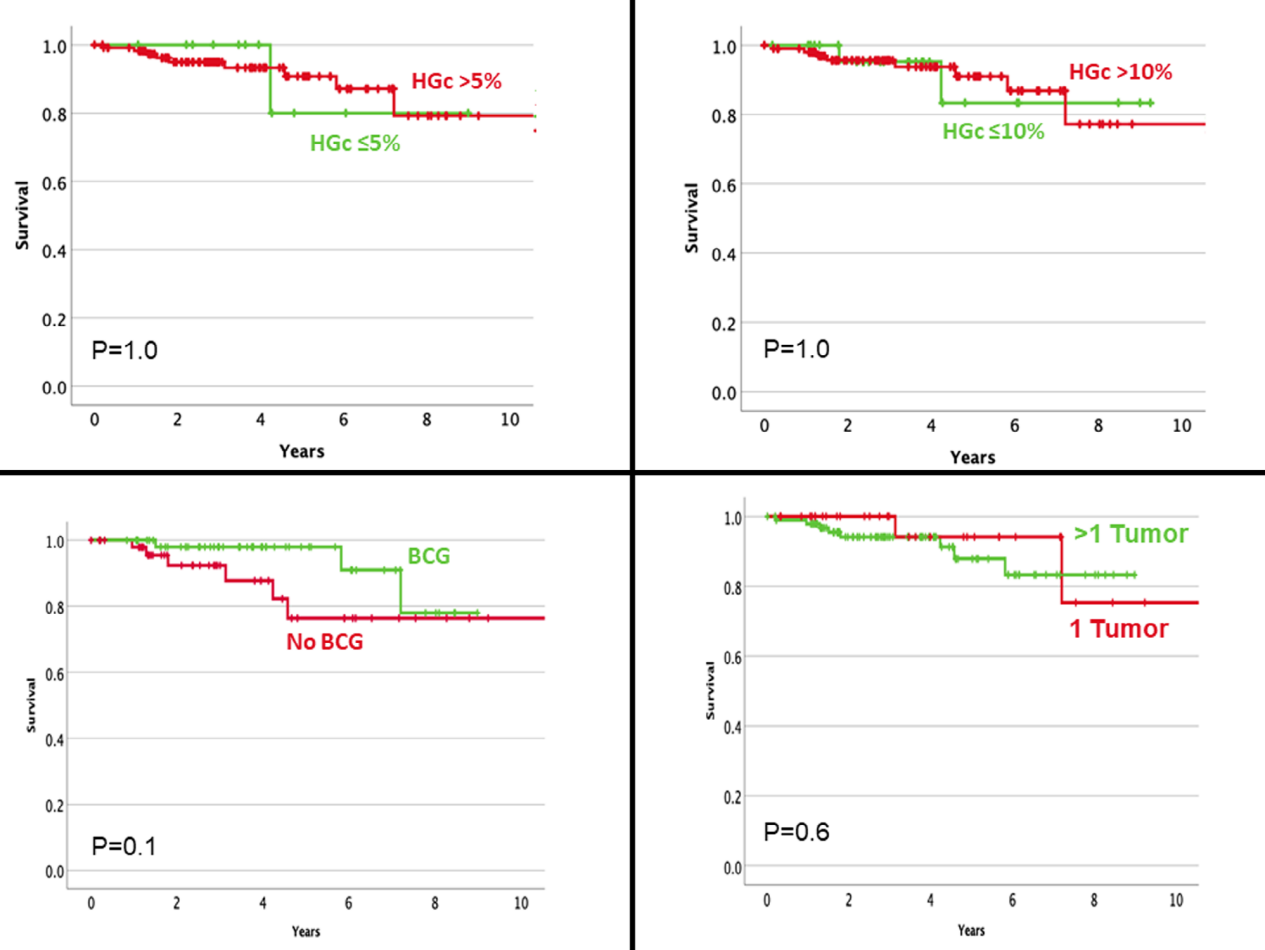
Kaplan–Meier curve illustrating the relationship between various clinical and pathological parameters and progression‐free survival. Non‐invasive papillary urothelial carcinomas with minor high‐grade component are not associated with a more indolent behaviour and should likely be regarded as high‐grade carcinomas.

Survival analysis of the pTaHG cohort revealed significant association between intravesical BCG, tumour multifocality, tumour size >3 cm and recurrence‐free survival while no histological parameter was significant (Figure 3). In terms of progression, no clinical nor histological feature was prognostic (Figure 4).

## Discussion

Our study explores the prognostic importance of the percentage of high‐grade component in patients with de novo non‐invasive urothelial papillary carcinoma, using strict inclusion criteria and detailed morphological evaluation. Unlike the recent WHO statement suggesting that tumours with minor HGc tend to behave more indolently, we found no significant differences in recurrence or progression for this subset of patients when compared to other pTaHGs. Patients with a minor HGc, defined as either ≤5% or ≤10%, showed significantly higher progression rates than pTaLG patients and comparable progression rates to other pTaHGs.

Currently, grading of non‐invasive urothelial papillary tumours is usually reported using either the WHO 1973 or the WHO 2004/2016 grading systems.^7^, ^8^, ^9^, ^10^ A recent survey among members of the European Association of Urology and the International Society of Urological Pathology found that 53% of respondents used only the WHO 2004 system, while 40% used both. Nearly half (48%) supported the introduction of hybrid WHO 1973/2004 grading systems.^13^, ^22^ The American Urological Association (AUA) endorses the 2004/2016 WHO grading systems for classifying NMIBC as low‐, intermediate‐ or high‐risk disease, recommending intravesical therapy for all intermediate‐ or high‐risk patients. In comparison, the European Association of Urology provides a risk calculator for NMIBC that incorporates either the 1973 or the 2004/2016 grading systems.^23^, ^24^ Notably, none of the international clinical guidelines have yet addressed the issue of minor HGc, and our findings actually argue against treating patients with minor HGc similar to pTaLGs. The only three parameters associated with recurrence were tumour multifocality, tumour size >3 cm and the lack of intravesical BCG therapy, regardless of percentage of HGc. Therefore, based on our results, patients with minor HGc should probably not be classified as low‐risk NMIBC and may be considered suitable candidates for intravesical BCG therapy.

Heterogeneity of grade in urothelial papillary tumours is common and occurs in up to 32% of tumours.^25^ Several previous studies have evaluated the behaviour of MGPUCs in comparison to low‐grade and high‐grade tumours, with conflicting results. The divergent outcomes reported in the literature are likely due to different inclusion criteria, with some studies involving all NMIBC cases, including invasive tumours (pT1), and others restricted to the non‐invasive category (pTa). Furthermore, while some studies, such as ours, focused solely on de‐novo cases, others included all pTaHG tumours, even if those tumours represented recurrences, which by definition put patients at a higher risk of further recurrence and progression. Additionally, most studies correlated outcomes with pure histological parameters without considering well‐established clinical prognosticators such as tumour multifocality, tumour size or intravesical BCG treatment. Histologically, different studies used variable cut‐offs of %HGc to define a tumour as MGPUC, making it difficult to compare the impact of HGc extent on prognosis accurately. Lastly, the non‐uniform treatment strategies and differing follow‐up periods also theoretically may contribute to these inconsistent results. Overall, the findings of these studies can be divided into those in which MGPUCs behaved similarly to pTaLGs, and those in which MGPUC outcomes were worse than pTaLGs, either similar to pTaHG or intermediate between pTaLGs and pTaHGs. A summary of major studies is included in Table 3.

**Table 3:** Literature review of major studies related to prognostic significance of high‐grade component in non‐muscle invasive urothelial carcinoma

	Year	Study	Numbers and methodology	Definition of HG	Mixed HG%	Pure HG%	Statistical method	Recurrence rate/progression rate	Survival
1	2000	Cheng *et al*.^25^	*n* = 164(all Ta). Scores of 1, 2, and 3. 1 = LMP 2 = LG 3 = HG Combining 1^ry^ grade and 2^ry^ grade, forming scores 2–6 Score 6 is HG >95%	Based on WHO/ISUP 1998: Loss of normal architecture, cell polarity, and cohesiveness, nuclear pleomorphism, hyperchromasia, prominent nucleoli, and frequent mitosis. Ignore minuscule areas of HG when assigning an overall grade	5–95	>95	Proportional hazards model	Recurrence: 41% Progression: 20%	PFS: Significant difference between score 5 and score 6 (HG >95%)
2	2013	Gofrit *et al*.^26^	LG = 454 HG = 156 MG = 32 Inclusion: Ta, T1 and Tis	NA	≤10	>10	Log rank test	Recurrence, Progression, Death (entire cohort): 36%, 6.4%, 3.6%	RFS: LG = MG = HG PFS (*≥*T2): HG < (LG = MG) DSS: HG < (LG = MG)
3	2014	Schubert *et al*.^27^	MG = 17 HG = 136 Inclusion: Ta or T1—all received BCG	NA	≤50	>50	Log rank test Multivariate analysis	NA	AIFS: HG<MG Pure HG is an independent risk for worse AIFS BCG response: Higher in MG
4	2015	Reis *et al*. 27	LG = 63 MG = 31 HG = 40	Architectural disorganization, nuclear enlargement, and pleomorphism, with increased mitotic activity	≤5	>5	Log rank test Multivariate analysis	Recurrence (LG, MG, HG): 53.8%, 45.2%, 36.1% Stage progression (LG, MG, HG): 6.7%, 0, 36.1% *(MG = LG) < HG*	PFS: *MG > HG* (unknown BCG status) Mortality: *HG > MG* (Not maintained upon adjusting for multifocality, size, IVT)
5	2021	Ho *et al*.^28^	LG = 127 MG = 27 HG = 66 Inclusion: Ta or T1 (62% HG).	NA	≤5/>5–80 (two different definitions)	>80	Multivariable Cox regression	Recurrence (LG, MG, HG): 41.7%, (78.6%/61.5%), 47% Stage progression (LG, MG, HG): 2.4%, 14.3%/15.4%, 4.5% *MG > (LG = HG)*	RFS: *LG = MG = HG*
6	2023	Kir *et al*.^20^	LG = 160 MG = 160 HG = 160 Inclusion: Ta, T1 and T2 and CIS	Architectural organization, nuclear size variation, hyperchromasia, irregular & pleomorphic nuclei, prominent nucleoli and increased mitotic activity. Conflicted samples were stained with Ki‐67 and percentage of each area was calculated, taking into account the intensely labelled regions with Ki‐67	<5	≥5	Log‐rank test Multivariate Cox regression	Recurrence (LG, MG, HG): 22.5%, 2.5%, 6.8% Stage progression (LG, MG, HG): 3.1%, 10%, 11% *(MG = HG) > LG*	RFS: *LG > MG* *MG > HG* DSS: *LG > MG* *MG > HG*
7	2023	Syed *et al*.^29^	G1 = 164 G2 = 17 G3 = 14 G4 = 78	NA	G2: ≤5 & G3: >5–25	G4: >25	Log‐rank test	Stage progression (G1,2,3,4): 6.7%, 23.5%, 28.6%, 41% *G4 > (G2 = G3) > G1* Metastasis (G1, 2, 3, 4): 5.5%, 5.9%, 7.1%, 17.9% *G4 > (G1 = G2 = G3)* Death (G1, 2, 3, 4): 4.3%, 5.9%, 7.1%, 15.4% *G4 > (G1 = G2 = G3)*	RFS*: G4 < (G1 = G2 = G3)* PFS: *G4 < (G2 = G3) < G1*
8	2025	Kir *et al*.^30^	Ta = 362	Same as study 6	<5 labelled CG1	≥5 Divided to CG2 (5–49), CG3 (50–99), CG4 (100)	Cox regression	Recurrence (LG, CG1, CG2, CG3, CG4): 28.6%, 48.5%, 42.9%, 44.1%, 57.5% Death (LG, CG1, CG2, CG3, CG4): 0.8%, 15.2%, 22.9%, 23.5%, 45.5% *CG4 (pure HG) > (CG2 = CG3) > CG1 > LG*	CSS (death or end of follow‐up): *LG>CG1 = CG2 = CG3 = CG4 (pure HG)*
9	2024	Khalatbari *et al*.^31^	LG = 59 MG = 41 HG = 50	NA	<5%	≥5%	Cox regression	Recurrence (LG, MG, HG): 55.9%, 34.2%, 56% Grade progression (LG, MG): 8.5%, 12.2% *MG>LG* Stage progression: (LG, MG, HG): 0, 0, 14%	RFS: *MG>LG>HG*
10	2025	Chambers *et al*.^32^	MG = 136 Inclusion: Ta and T1 (bladder and ureter)	NA	≤5%	—	—	Recurrence: 52% Stage progression: 1.8%	NA
11	2025	Mi *et al*.^21^	LG = 52 MG = 52 HG = 52	Severe cytologic atypia with nuclear enlargement, hyperchromasia, pleomorphism, and prominent nucle	≤5%	≥5%	Log‐rank test Cox regression	Recurrence (LG, MG, HG): 67%, 52%, 68% Stage progression (LG, MG, HG): 0, 6%, 20% *HG>MG>LG*	Recurrence probability: *LG = MG = HG* Stage progression probability: *HG>MG>LG*

AIFS: advanced intervention‐free survival; CIS: carcinoma in situ; CSS: Cancer‐specific survival; DSS: Disease specific survival; IVT: intravesical therapy; LMP: Low malignant potential; PFS Progression‐free survival; RFS recurrence‐free survival.

One of the earliest studies to account on MPUC was by Cheng *et al*., where different areas of tumour cell atypia within a tumour were assigned different grades (grade 1, 2, or 3), and a combined score of the primary and secondary grades was calculated for each case.^25^ Cases with a combined grade of 5 (corresponding to MGPUC) showed progression rates intermediate between grade 4 (pure pTaLG) and grade 6 (pure pTaHG) tumours. In that study, minute areas (defined as<5%) of HGc were ignored and not incorporated to the final grade, making it impossible to compare the behaviour of tumours with minor HGc to the rest of the cohort. Gofrit *et al*., compared the outcome of 32 patients with MGPUC to 454 pTaLGs and 156 pTaHGs.^26^ Tumours with ≤10% HGc were classified as MGPUC, whereas those with >10% HGc were labelled as pTaHG. In that series, progression‐free survival and disease‐specific survival were similar for pTaLGs and MGPUCs but significantly lower than for patients with pTaHGs. In the Reis *et al*. study, where MGPUC was defined as HGc ≤5%, 42.5% of MGPUCs recurred and none progressed in stage.^33^ The overall prognosis of those tumours, in terms of stage progression and survival, was closer to pTaLGs than pTaHGs. However, BCG‐untreated MGPUC showed significantly higher grades of progression compared to pTaLGs, leading the authors to argue for keeping cases with HGc ≤5% as a distinct subcategory. In *Chambers et al*.'s study, 136 patients with MGPUC (defined as ≤5% HGc) pTa/pT1 bladder and ureteral tumours were included.^32^ In that study, 52.2% recurred, usually as pTaLG/MGPUC, 13.2% progressed to pTaHG/pT1HG, 1.8% showed stage progression from pTa to pT1, and none showed stage progression to pT2 disease. The authors concluded that this behaviour, which is similar to pTaLG, justifies considering pTa tumours with ≤5% HGc as a distinct subcategory with a better prognosis than other pTaHGs. However, that study included tumours of different stages (pTa + pT1) as well as upper tract tumours, which are different from a biological and therapeutic standpoints from bladder tumours. *Schubert et al*. assessed BCG response in 17 MGPUCs compared to 136 NMIBC HG tumours.^27^ In that study, MGPUC, defined as tumours with <50% HGc, responded significantly better than HG tumours, suggesting a biologically more indolent disease. As treatment response was the end‐point in this study, it did not include pTaLG tumours and a comparison to MGPUC cannot be made in terms of recurrence and progression. Moreover, this study included all NMIBC cases including pT1. Syed *et al*. studied 273 pTas and divided them into four grades (G1‐G4) based on %HGc (using 0%–5%–25% cut‐offs).^29^ While there was no difference in recurrence among the groups, G2 and G3 tumours exhibited intermediate prognosis for grade and stage progression compared to G1 and G4. Interestingly, the prognostically significant cutoff for HGc was 25% rather than 5%, as G2 and G3 tumours behaved similarly. In another study by Kir *et al*., MGPUC (defined as HGc≤5%) showed significantly worse recurrence and stage progression rates than pTaLG, but comparable rates to pTaHG (defined as tumours with >5% HGc).^20^ In that study, Ki67 immunostaining was used to separate the low‐grade from high‐grade components in difficult cases. It is unclear however in how many cases this approach was used, as it is not standard practice and does not align with the latest GUPS recommendations of not relying on tissue biomarkers in grading urothelial carcinomas.^19^ Subsequent study from the same group showed significant lower cancer specific survivals for patients with pTa tumours with a HGc in comparison to TaLGs, and this is independent of the %HGc.^31^ In that study, the %HGc did not influence outcome. The most recent study on this subject was by Mi *et al*., who compared the outcomes of three cohorts: 52 MGPUCs (HGc ≤5%), 52 pTaLGs and 52 pTaHGs. While no significant difference in recurrence rates was observed among the three cohorts, MGPUCs exhibited stage progression rates (6%) that were intermediate between pTaLGs (0%) and pTaHGs (20%).^21^

The molecular landscape of the MGPUC category has only rarely been evaluated. Downes *et al*. assessed FGFR3 mutations and CDKN2A deletions using fluorescence in‐situ hybridisation in 29 MHPUCs (defined as HGc <20%) and 8 pTaLGs. A homozygous CDKN2A deletion was identified in the low‐grade areas of 88% of FGFR3 mutant cases (including five pTa), in five of nine FGFR3 wild‐type carcinomas and in none of the pTaLGs. The presence of homozygous CDKN2A deletions, a feature often seen in high‐grade carcinomas and linked to stage progression, in the low‐grade areas of MGPUC, indicated that these deletions occur before morphological changes can be detected microscopically. Consequently, pTa tumours with mixed low‐ and high‐grade areas are likely better regarded as high‐grade at the molecular level.^34^, ^35^ Taking this observation into account and perceiving the variability among different studies' design, inclusion criteria and findings, as well as the lack of control for many clinical factors influencing outcome in most studies, the question of whether there are sufficiently solid data to justify potentially including patients with MGPUC and minor HGc in the low‐risk NMIBC category should be raised. This question is even more legitimate in the context where the majority of studies show higher rates of stage progression in MGPUC with minor HGc in comparison to pTaLG cases and rare studies showing comparable rates to pTaHGs, including ours. Since the practical clinical question is whether those patients should be offered adjuvant therapy, it is difficult in our view to argue that such cases should clinically be managed as pTaLGs. Finally, an unaddressed factor influencing the comparison of different studies using strict quantitative cut‐offs is the inherent inter‐observer diagnostic variability among pathologists when diagnosing a high‐grade component, especially in grey‐zone and borderline cases. In our practice, we tend to rely mainly on nucleomegaly (4–5 times the size of lymphocytes), severe hyperchromasia, and pleomorphism visible at low to intermediate magnification as objective, consistent, and reliable indicators of high‐grade areas, in an approach analogous to the Paris system for reporting urine cytology.^36^ While other features such as loss of polarity, prominent nucleoli, mitotic activity are listed in the WHO criteria of high‐grade papillary lesions, these criteria are presented without guidance on their required frequency, relative weighting or whether any of them are mandatory. Moreover, several descriptors, such as ‘rare versus frequent’ mitoses are inherently subjective, which likely contributes to the known interobserver variability in grading. As shown in our data, the presence cytological features other than nucleomegaly, severe hyperchromasia and pleomorphism varies greatly, with necrosis only present in 14% of our pTaHG cohort and the highest number of mitoses per 1 HPF ranging from 1 to 18. The same variability is expected for architectural disarray, prominent nucleoli and cellular discohesion, which are inconsistently observed in pTaHGs in routine clinical practice. Therefore, ongoing efforts should be made to refine the histological criteria used for grading through objective and reproducible descriptions. There is also a need for the official adoption of illustrative figures that highlight the spectrum of low‐grade and high‐grade areas, which pathologists can refer to in their daily practice. In addition, advances in digital pathology and artificial intelligence are poised to improve the consistency of histologic grading by reducing subjective variability and enabling quantitative assessment of morphologic features. As these technologies mature, they may help overcome the limitations imposed by intratumoral heterogeneity and enhance the predictive accuracy of current risk models, especially in studies in which only a single pathologist reviews the cases, by providing standardized, quantitative metrics that complement traditional morphology and enhance overall reproducibility. Of note is that even a very small number of unequivocally high‐grade cells could theoretically qualify a tumour as HG, in routine practice it is extraordinarily uncommon to encounter only isolated high‐grade cells, and grading is instead based on a discernible, albeit small, high‐grade component that is discernible at low‐ to intermediate‐ magnification.

The strengths of the current study are the fact that it only included de‐novo cases, the detailed histological assessment and the incorporation of additional clinical prognostic factors making it possible to evaluate the independent and additive prognostic value of the %HGc element. The limitations of the study include its retrospective nature, the relatively small cohort's size, the small number of cases with minor HGc, the relatively short follow‐up period as well as an enrichment for the intermediate‐risk pTaHG category (mostly unifocal tumours smaller than 3 cm in size).

In summary, we believe that, based on our results and the inconsistent and inconclusive literature findings, patients with non‐invasive papillary urothelial tumours containing a minor high‐grade component should not be considered analogous to pTaLGs and should likely be offered adjuvant therapy.

## Author contributions

Study coordination and design, data collection, data analysis, writing of manuscript, approval of final version: M.S.F.; Data collection, critical review of the manuscript, approval of final version: N.O. and J.H.; Data analysis, foundation model algorithm, review of the manuscript, approval of final version: A.L.; Study coordination and design, data collection, review of the manuscript, approval of final version: W.K.; Study coordination and design, data collection, review of the manuscript, approval of final version: F.B.

## Conflict of interest

The authors report no conflict of interest.

## Data Availability

Individual participant data will not be shared.
